# Beyond the Usual Pathogens: Community-Acquired Meningoencephalitis Caused by Streptococcus equi in an Immunocompetent Adult

**DOI:** 10.7759/cureus.114847

**Published:** 2026-08-20

**Authors:** Hicham Hammadi, Anas Elbouti, Brahim Chikhi, Youssef Aarjouni, Hamza Madouim, Mohamed Ezzaim, Gabriel Edudzi, Noureddine Kartite, Abdelhafid Houba, Bakkali Hicham, Nawfal Doghmi

**Affiliations:** 1 Anesthesia and Critical Care, Mohammed V Military Training Hospital, Rabat, MAR

**Keywords:** cerebrospinal fluid, meningoencephalitis, status epilepticus, streptococcus equi, zoonotic infection

## Abstract

*Streptococcus equi* is a rare zoonotic pathogen that only occasionally causes invasive central nervous system infections in humans. We report the case of a previously healthy 50-year-old man who presented with fever and altered mental status in the setting of recent animal exposure. Cerebrospinal fluid analysis was consistent with acute bacterial meningitis, while direct microscopy and multiplex polymerase chain reaction identified *S. equi*. Antimicrobial susceptibility testing confirmed susceptibility to ceftriaxone. Despite early initiation of intravenous ceftriaxone and adjunctive dexamethasone, the patient's course was complicated by generalized status epilepticus requiring intensive care support. He subsequently showed progressive neurological recovery with a favorable outcome. This case underscores the importance of considering *S. equi* as a potential cause of community-acquired bacterial meningoencephalitis in patients with relevant zoonotic exposure and highlights the value of rapid molecular diagnostics for early pathogen identification and targeted antimicrobial therapy.

## Introduction

Acute bacterial meningoencephalitis is a medical emergency associated with substantial morbidity and mortality despite advances in antimicrobial therapy and intensive care. While *Streptococcus pneumoniae* and *Neisseria meningitidis* remain the leading causative pathogens in immunocompetent adults, infections caused by uncommon zoonotic bacteria are rare and may delay diagnosis and appropriate management [1,2].

*Streptococcus equi* is a zoonotic, β-hemolytic streptococcus belonging to Lancefield group C. The species comprises three recognized subspecies: *S. equi* subsp. *equi*, *S. equi* subsp. *zooepidemicus*, and *S. equi* subsp. *ruminatorum*. It primarily infects animals, particularly horses, while *S. equi* subsp. *zooepidemicus* may also cause infections in other domestic animals, including cattle, sheep, goats, pigs, dogs, and cats. Although human infections are rare, *S. equi* subsp. *zooepidemicus* has been reported as a zoonotic pathogen capable of causing invasive disease in humans. Human infections have been associated with close contact with infected animals or exposure to contaminated animal-derived products and may present as bacteremia, endocarditis, septic arthritis, or, exceptionally, central nervous system infection [3,4].

Because of its rarity, *S. equi* meningoencephalitis remains poorly described in the literature. We report a case of community-acquired meningoencephalitis caused by *S. equi* in a previously healthy, immunocompetent adult. This case highlights the importance of considering uncommon zoonotic pathogens in patients presenting with acute bacterial meningoencephalitis and underscores the value of rapid microbiological diagnosis in facilitating timely, targeted antimicrobial therapy.

## Case presentation

A 50-year-old man with no significant past medical history was admitted to the intensive care unit because of febrile altered mental status. He had no known history of chronic disease or immunocompromising condition and was not receiving immunosuppressive therapy.

His illness began two days before admission with a flu-like syndrome characterized by fever and asthenia, followed by progressive deterioration of consciousness. History taking revealed recent contact with a newly adopted dog, raising the possibility of zoonotic exposure.

On admission, neurological examination revealed an unconscious patient with a Glasgow Coma Scale (GCS) score of 10/15, with spontaneous eye opening (E4), no verbal response (V1), and localization of pain (M5). The pupils were equal and reactive to light. No clinical seizures were observed. Brainstem reflexes were preserved, and nuchal rigidity was present. The patient was hemodynamically and respiratory stable.

Initial laboratory investigations (Table 1) demonstrated a normal blood glucose level, marked inflammatory syndrome with a C-reactive protein level of 460 mg/L, a procalcitonin level of 7.38 ng/mL, and leukocytosis of 23,000 cells/mm^3^ with neutrophil predominance. Serum electrolytes, calcium-phosphate balance, and the remainder of the biochemical workup were unremarkable. HIV serology was negative.

**Table 1:** Laboratory findings on admission AST: aspartate aminotransferase; ALT: alanine aminotransferase

Laboratory parameter	Patient value	Reference range
White blood cell count	23,000 cells/mm^3^	4,000-10,000 cells/mm^3^
Neutrophils	20000 cells/mm^3^	1500-7500 cells/mm^3^
C-reactive protein	460 mg/L	<5 mg/L
Procalcitonin	7.38 ng/mL	<0.5 ng/mL
Blood glucose	1.02 g/L	0.70-1.10 g/L
Sodium	143 mmol/L	135-145 mmol/L
Potassium	3.8 mmol/L	3.5-5.0 mmol/L
Chloride	100 mmol/L	98-106 mmol/L
Bicarbonate	25 mmol/L	22-29 mmol/L
Calcium, total	2.3 mmol/L	2.15-2.55 mmol/L
Phosphate	1.3 mmol/L	0.80-1.50 mmol/L
Magnesium	0.9 mmol/L	0.70-1.00 mmol/L
Albumin	38 g/L	35-50 g/L
Creatinine	71 µmol/L	60-110 µmol/L
Urea	5.2 mmol/L	2.5-7.5 mmol/L
AST	35 U/L	<40 U/L
ALT	38 U/L	<40 U/L

Emergency non-contrast brain computed tomography (CT) revealed no abnormalities. Cerebrospinal fluid (CSF) analysis showed 740 leukocytes/mm^3^ (72% neutrophils), 210 red blood cells/mm^3^, elevated protein concentration (2.82 g/L), and low glucose concentration (0.32 g/L), with a simultaneous serum glucose level of 1.02 g/L. Direct examination of the CSF was followed by bacterial culture, which yielded abundant growth of *S. equi*. The isolate was identified by matrix-assisted laser desorption/ionization time-of-flight mass spectrometry (MALDI-TOF MS), and multiplex polymerase chain reaction (PCR) performed on the CSF also detected *S. equi*. Antimicrobial susceptibility testing demonstrated susceptibility to ceftriaxone and the other tested antibiotics, except for tetracycline, to which the isolate was resistant.

Empirical antimicrobial therapy was initiated immediately with high-dose intravenous ceftriaxone (3 g every 12 hours), combined with adjunctive dexamethasone (10 mg every six hours), together with standard intensive care supportive measures and close neurological monitoring.

During hospitalization, the patient developed generalized status epilepticus requiring endotracheal intubation, mechanical ventilation, continuous intravenous sedation, and antiepileptic treatment with levetiracetam (500 mg every eight hours). Following clinical stabilization, sedation was gradually discontinued. A follow-up electroencephalogram (EEG) showed no evidence of ongoing epileptic activity. The patient was successfully extubated after fulfilling standard weaning criteria. Following extubation, neurological examination revealed complete recovery of consciousness (GCS 15/15), although left-sided hemiparesis persisted.

**Figure 1:**
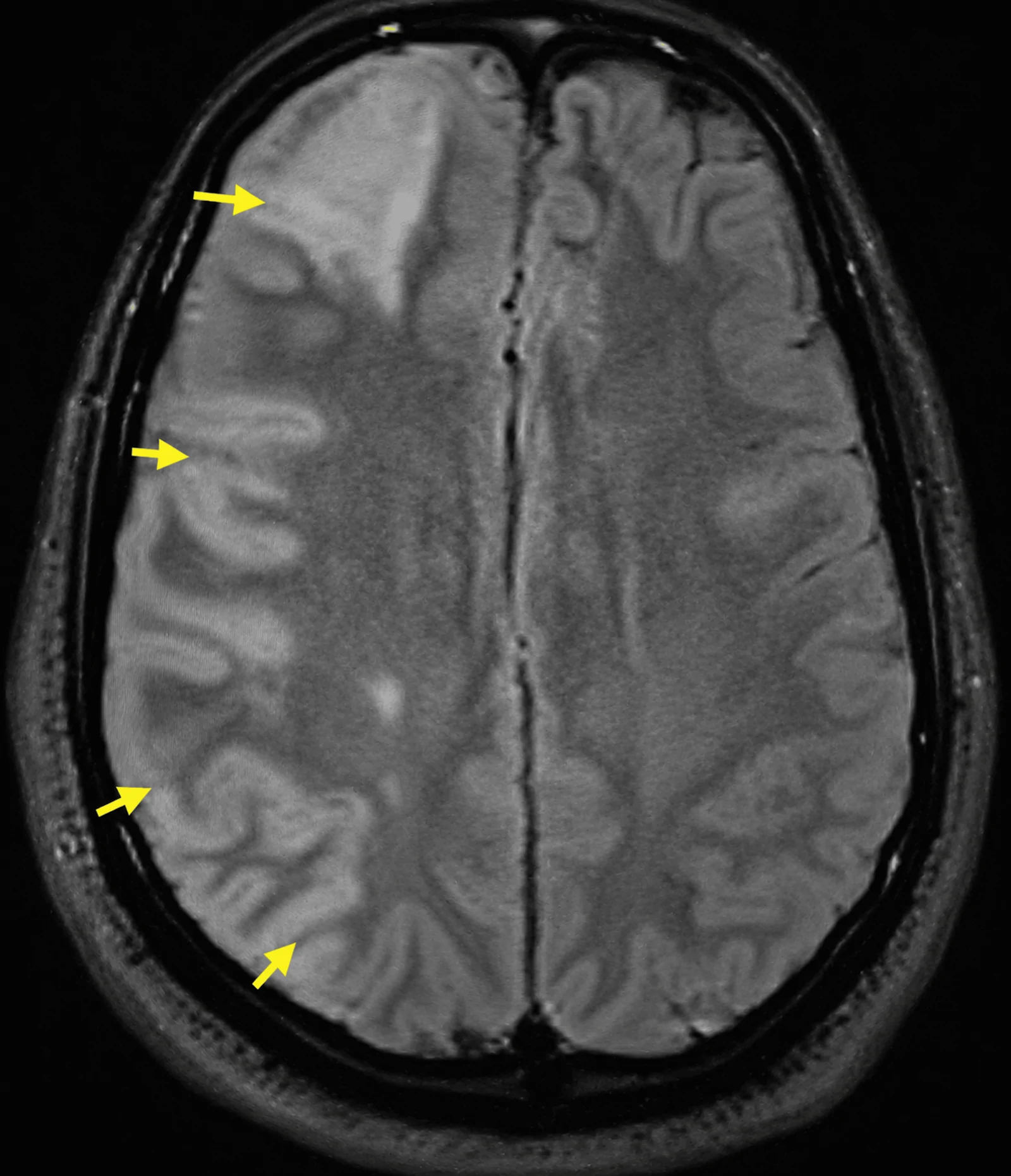
Axial FLAIR MRI showing right frontoparietal cortical and subcortical hyperintensities consistent with infectious meningoencephalitis (yellow arrows) The FLAIR hyperintense abnormalities involve the right frontoparietal cortex and adjacent subcortical white matter (yellow arrows), reflecting parenchymal involvement in the setting of infectious meningoencephalitis. FLAIR: fluid-attenuated inversion recovery

**Figure 2:**
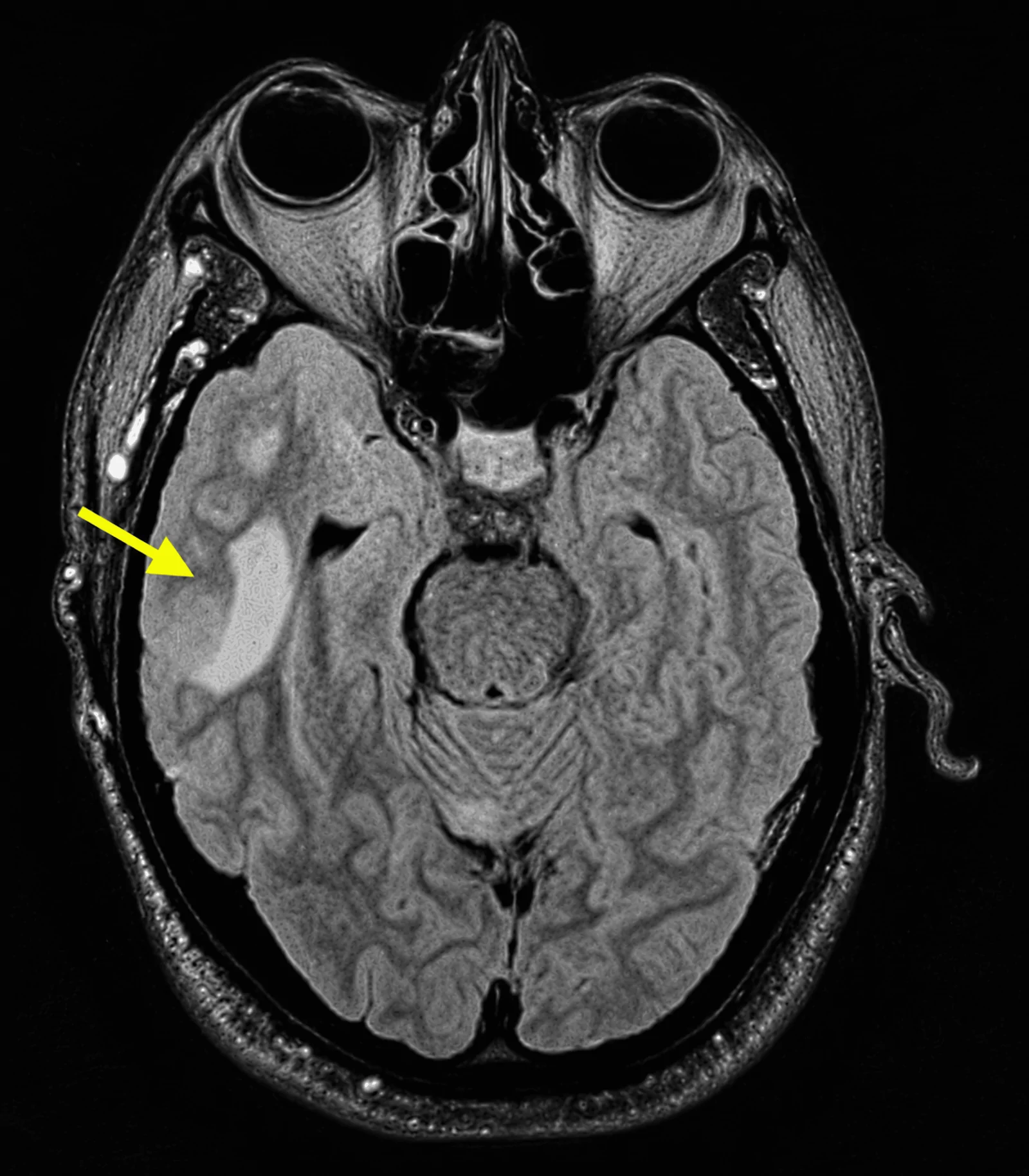
Axial FLAIR MRI showing right temporal cortical and subcortical hyperintensities consistent with infectious meningoencephalitis (yellow arrow) The FLAIR hyperintense abnormalities involve the right temporal cortex and adjacent subcortical white matter (yellow arrow), demonstrating the temporal distribution of the abnormalities associated with infectious meningoencephalitis. FLAIR: fluid-attenuated inversion recovery

Brain magnetic resonance imaging (MRI) (Figures 1-2) demonstrated findings consistent with infectious meningoencephalitis, including extensive cortical and subcortical T2-weighted and fluid-attenuated inversion recovery (FLAIR) hyperintensities involving the right frontal, parietal, temporal, and insular lobes, with extension to the midbrain and ipsilateral cerebellar hemisphere. No diffusion restriction was observed. Post-gadolinium sequences demonstrated gyriform cortical enhancement and diffuse leptomeningeal enhancement, more prominent over the right frontal region.

The patient completed a 14-day course of high-dose ceftriaxone and four days of dexamethasone. His clinical course was favorable, with complete recovery of consciousness and progressive improvement of the residual neurological deficit.

## Discussion

Human meningitis caused by *S. equi *is an exceptionally rare zoonotic infection that can result in severe neurological complications and potentially fatal outcomes [5,6]. *S. equi* is a Gram-positive, β-hemolytic, Lancefield group C streptococcus primarily recognized as an equine pathogen. Human infection is uncommon and has most often been associated with close contact with horses or other animals, as well as the consumption of unpasteurized animal products [5,6]. In our patient, recent contact with a newly adopted dog represents a relevant epidemiological clue. Although the animal was not microbiologically investigated and a direct zoonotic transmission cannot therefore be established, canine-associated transmission of *S. equi* has previously been reported in human meningitis [7].

Meningitis caused by *S. equi* is associated with a poor prognosis, with a reported mortality rate of approximately 24%. Among survivors, complete neurological recovery has been reported in only 38% of cases, highlighting the substantial burden of long-term neurological sequelae despite appropriate treatment [3]. CSF examination usually reveals the typical biochemical and cytological features of bacterial meningitis, including neutrophilic pleocytosis, low glucose concentration, and elevated protein levels. Gram staining is positive for Gram-positive cocci in approximately 50% of cases [6].

In our patient, microbiological investigation of the CSF included direct examination, bacterial culture, and molecular testing. CSF culture was positive, with abundant growth of *S. equi*. The isolate was further identified by MALDI-TOF MS, confirming the identification of *S. equi.* A multiplex PCR assay performed on the CSF also detected *S. equi*. Antimicrobial susceptibility testing showed that the isolate was susceptible to ceftriaxone, penicillin G, ampicillin, gentamicin, chloramphenicol, tigecycline, erythromycin, lincomycin, clindamycin, linezolid, vancomycin, teicoplanin, trimethoprim-sulfamethoxazole, ciprofloxacin, levofloxacin, moxifloxacin, norfloxacin, and rifampicin, while resistance to tetracycline was observed. The concordant culture, MALDI-TOF MS identification, and molecular findings supported the diagnosis of *S. equi* meningoencephalitis and guided targeted antimicrobial therapy.

Approximately half of patients with *S. equi* meningitis develop complications. The ability of the organism to produce extracellular hyaluronate lyase, an important virulence factor facilitating tissue invasion and bacterial spread, may contribute to the severity of the infection [8]. Severe neurological complications, including cerebral infarction, subdural empyema, hearing impairment, and persistent neurological deficits, have been reported in previous cases [6,7,9]. Our patient developed generalized status epilepticus requiring mechanical ventilation and intensive care management.

Because of the rarity of invasive *S. equi *infections, no pathogen-specific therapeutic guidelines are currently available. Management therefore relies on contemporary recommendations for acute bacterial meningitis. Current international guidelines recommend the prompt initiation of empiric intravenous antimicrobial therapy with a third-generation cephalosporin, such as ceftriaxone or cefotaxime, with subsequent de-escalation based on microbiological identification and antimicrobial susceptibility testing [10,11]. Most reported *S. equi* isolates remain susceptible to β-lactam antibiotics, including penicillin and third-generation cephalosporins [12]. In our patient, antimicrobial susceptibility testing confirmed susceptibility to ceftriaxone, supporting continuation of targeted ceftriaxone therapy.

Adjunctive dexamethasone should be administered immediately before or concomitantly with the first dose of antibiotics to attenuate the inflammatory response and reduce the risk of neurological complications in patients with suspected acute bacterial meningitis [10,11]. In addition to targeted antimicrobial therapy, optimal management requires comprehensive supportive care, including intensive care monitoring, airway protection, seizure control, and mechanical ventilation when indicated. Our patient received 14 days of high-dose ceftriaxone and four days of dexamethasone, with progressive neurological improvement and a favorable outcome.

## Conclusions

*S. equi* should be considered a rare cause of community-acquired bacterial meningoencephalitis, particularly in patients with recent animal exposure. In this case, the diagnosis was supported by positive CSF culture, MALDI-TOF MS identification, and multiplex PCR detection. This case highlights the importance of a detailed exposure history and comprehensive microbiological investigation, combining conventional culture, reliable identification methods, and molecular testing, to facilitate timely diagnosis and targeted antimicrobial therapy.
